# A Bioactive Benzyl Terpene from *Acridocarpus smeathmannii* Inhibits Human Prostate Smooth Muscle Contractility

**DOI:** 10.3390/molecules31091380

**Published:** 2026-04-22

**Authors:** Oluwafemi Ezekiel Kale, Claudia Huber, Denis Schuldeis, Alexander Tamalunas, Martin Hennenberg, Wolfgang Eisenreich

**Affiliations:** 1 Department of Pharmacology and Therapeutics, Olabisi Onabanjo University, Ago-Iwoye 120101, Ogun, Nigeria; 2 Bavarian NMR Center (BNMRZ), School of Natural Sciences, Technical University of Munich, 80333 München, Germany; claudia.huber@tum.de (C.H.); denis.schuldeis@tum.de (D.S.); 3 Urologische Klinik und Poliklinik LMU Klinikum, LMU München, 80539 München, Germany; alex.tamalunas@web.de (A.T.); martin.hennenberg@med.uni-muenchen.de (M.H.)

**Keywords:** 2-(5-Isopropyl-4-methoxy-2-methylbenzyl)phenol, chamanen, CAS Registry Number: 64421-22-3, chemical characterization, anticontractility study, toxicity effect, *Acridocarpus smeathmannii* roots

## Abstract

The roots of *Acridocarpus smeathmannii* were identified as a natural source of the benzyl-terpene 2-(5-isopropyl-4-methoxy-2-methylbenzyl)phenol (FAH-01, chamanen), which was isolated and structurally characterized by chromatographic and spectroscopic techniques, including two-dimensional NMR analysis. Functionally, FAH-01 exerted pronounced inhibitory effects on human prostate smooth muscle contractility. In organ bath experiments, it reduced noradrenaline-induced contractions by up to 72% and phenylephrine-induced contractions by up to 63%, without affecting agonist potency (pEC_50_). During electrical field stimulation (2–32 Hz), FAH-01 suppressed neurogenic contractile responses, indicating interference with adrenergic and nerve-mediated signaling pathways. Beyond smooth muscle modulation, FAH-01 showed antioxidant activity in the DPPH radical-scavenging assay and exhibited early-stage toxicity in the *Artemia salina* cysts. Collectively, these findings identify FAH-01 as a bioactive natural product with potent inhibitory effects on adrenergic and neurogenic contraction in human prostate smooth muscle, supporting its therapeutic potential in conditions associated with increased smooth muscle tone. Further preclinical studies are needed to elucidate its mechanisms of action, toxicity, and in vivo efficacy.

## 1. Introduction

Recent advances in phytotherapy and natural product research highlight the importance of isolating and purifying plant-derived metabolites to elucidate their biological activities and assess their potential in drug discovery and development [1]. Despite substantial progress in analytical, physical, and applied chemistry, the comprehensive structural characterization of plant secondary metabolites and the biosynthetic diversity that is inherent to medicinal plants remain insufficiently explored [2]. Addressing this gap is critical for linking chemical structure to biological function.

*Acridocarpus smeathmannii* (DC.) Guill. & Perr. is a plant species whose roots have been documented in ethnobotanical surveys [3]. Although its ethnopharmacological use is relatively limited, *A. smeathmannii* has been employed in traditional African medicine, particularly in the management of male reproductive and related disorders.

Reports on the biochemical and pharmacological significance of *Acridocarpus smeathmannii* demonstrate its ability to inhibit human prostate and bladder smooth muscle contraction, as well as suppress the proliferation and viability of prostate stromal cells [4]. These findings support its traditional use in the management of male reproductive disorders and suggest a direct modulatory effect on prostate physiology. Also, experimental evidence shows that the extract can reduce α_1_-adrenergic- and cholinergic-induced contractions, indicating interference with key pathways regulating lower urinary tract function. In addition, its cytotoxic and antiproliferative effects on stromal cells resemble mechanisms targeted by conventional therapies for benign prostatic hyperplasia (BPH) [4]. The discovery of novel bioactive compounds increasingly relies on integrated approaches combining preparative chromatographic separation with advanced spectroscopic analyses. Our group recently identified benzyl benzoate as one of the most abundant constituents in *A. smeathmannii* roots, a compound known for its vasodilatory properties [5]. Building on this work, we isolated, purified, and characterized 2-(5-isopropyl-4-methoxy-2-methylbenzyl)phenol (FAH-01), a benzyl terpene, by using preparative thin-layer chromatography, repeated column chromatography, reverse-phase high-performance liquid chromatography, gas chromatography–mass spectrometry, and comprehensive one- and two-dimensional NMR spectroscopy, with confirmation of the molecular formula by high-resolution mass spectrometry.

Structurally, FAH-01 represents a covalently linked benzyl–terpene hybrid, in which an aryl-methylene (benzyl) moiety is directly fused to a terpene scaffold. The compound was first isolated from the root bark of the plant *Uvaria chamae* and named chamanen [6]. Later, the same compound was isolated from the roots of *Xylopia africana* [7]. The hybrid architecture of FAH-01 integrates two complementary pharmacophoric motifs within a single molecule. Terpene scaffolds are recognized for their structural diversity, lipophilicity, and ability to interact with biological targets through defined three-dimensional conformations [8,9]. In parallel, benzyl and aromatic moieties confer π-π stacking potential and hydrogen-bonding interactions that can enhance molecular recognition and receptor binding [10,11]. The convergence of these features puts FAH-01 within a distinct region of chemical space with potentially improved drug-like properties, relative to its individual structural components.

Hybrid molecules incorporating multiple bioactive scaffolds have increasingly been recognized for their capacity to elicit dual or enhanced pharmacological effects [12,13]. Terpenes and benzyl derivatives exhibit well-established antispasmodic properties [5,14,15]. This concept is particularly relevant for FAH-01, as terpenes are widely associated with diverse biological activities, while benzyl-derived structures are valued for their chemical stability and interaction potential with biological targets [16]. The integration of these motifs within a single molecular framework may confer synergistic advantages in biological performance.

In the present study, prostate smooth muscle was selected as the experimental model due to its central role in the adrenergic regulation of urinary function. This choice was further supported by the structural characteristics of FAH-01, whose lipophilic benzyl–terpene architecture suggests a potential for interaction with smooth muscle receptors and ion channels. It is hypothesized that the hybrid benzyl–terpene structure of FAH-01 enables simultaneous interactions with receptor binding domains and lipid membranes, thereby facilitating both receptor-level antagonism and modulation of ion channel activity. 

Despite these considerations, detailed structural and pharmacological investigations of FAH-01 remained scarce. Therefore, the study was designed with two primary objectives: first, to provide a more comprehensive structural characterization of FAH-01 (chamanen) with in-depth NMR and MS analysis; and second, to evaluate its preliminary effects on smooth muscle contractility using agonist-induced contractions in human prostate tissue obtained following prostatectomy. Additionally, the acute toxicity profile of FAH-01 using *Artemia salina* bioassays and in vitro antioxidant capacity were assessed.

## 2. Results

### 2.1. Structural Analysis of 2-(5-Isopropyl-4-methoxy-2-methylbenzyl)phenol (FAH-01, Chamanen)

The liquid–liquid fractionation of the *n*-hexane extract was performed using a dichloromethane–water system (3:5, *v*/*v*) with distilled water. The aqueous phase (part A) was collected and dried under reduced pressure at 40 ± 1 °C and 72 mbar. Part A was subsequently dissolved in ethyl acetate (EtOAc) and washed three times with distilled water (1:1.5, *v*/*v*).

Combined fractions from Part A were initially screened using an n-hexane-ethyl acetate (9:1, *v*/*v*) solvent system. For preparative separation, fractions 33–39 were subjected to column chromatography using a 20 cm × 5 cm glass column packed with silica gel 60. Elution was performed isocratically with *n*-hexane-ethyl acetate (9:1, *v*/*v*) at a flow rate of 2.5 mL/min. A total of 119 fractions (4 mL each) were collected and monitored by preparative TLC, using the same solvent system.

**Figure 1:**
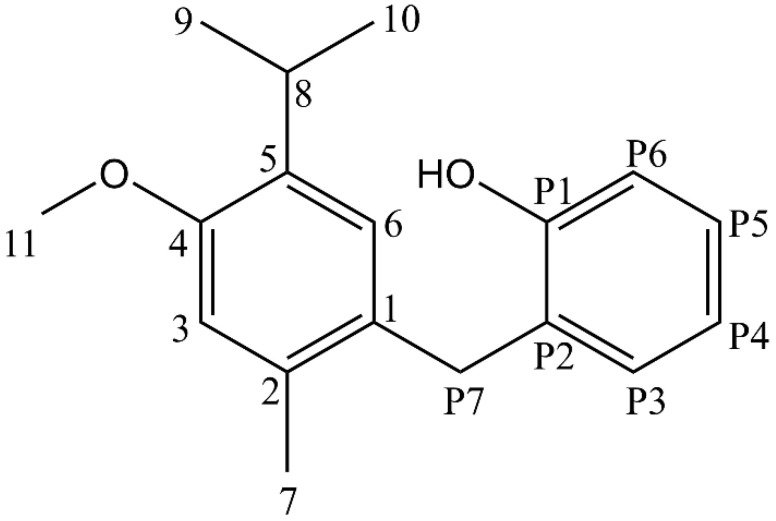
Chemical structure of 2-(5-isopropyl-4-methoxy-2-methylbenzyl)phenol (FAH-01, chamanen) with carbon atom positions as assigned in Table 1.

Fractions exhibiting identical R_f_ values were pooled and concentrated under reduced pressure using a rotary evaporator. Further purification was achieved by repeated column chromatography, yielding compound FAH-01 as a benzyl-terpenoid fraction (fractions, 86 to 106 (20 mg each) amounting to 0.4 g, reflecting its occurrence as a minor secondary metabolite, see Figure S5). FAH-01 (Figure 1) was obtained as a golden liquid that crystallized upon standing.

Gas chromatographic analysis of FAH-01 also revealed a single peak with a retention time of 22.64 min (Figure S1), indicating high purity (>95% FAH-01). The corresponding mass spectrum exhibited a molecular ion peak [M]^+^ at m/z 270.16 (Figure S2), consistent with the proposed molecular formula C_18_H_22_O_2_, and a base peak at m/z 107. A similarity search against the NIST mass spectral library yielded no positive match. The GC-MS results and suggested fragmentations are shown in Figures S1 and S2. High-resolution mass spectrometry (HRMS) resulted in an exact molecular mass of 270.1614 (Figure S3), further supporting the molecular formula of C_18_H_22_O_2_. Reversed-phase HPLC analysis of the FAH-01 fraction showed a single peak (99.8% FAH-01; Figure S4) with a retention volume of 28.47 mL. FAH-01 has a R_f_ value of 0.27 on preparative TLC (n-hexane-ethyl acetate, 9:1, *v*/*v*) (Figure S5).

Detailed structural elucidation was achieved by NMR spectroscopy. The ^1^H NMR spectrum (500 MHz, CDCl_3_; Figure S6, Table 1) displayed characteristic proton resonances between δ 6.93 and 7.11 ppm, appearing as doublets and triplets with a coupling constant of 7.7 Hz, which was indicative of a substituted aromatic ring system. The methyl doublet at δ 1.15 ppm (J = 6.5 Hz, relative intagral of 6) was consistent with the methyl groups of an isopropyl substituent. Additional singlet resonances were observed at δ 3.91 (2H), 6.68 (1H), 6.96 (1H), 2.22 (3H), and 3.81 ppm (3H), corresponding to methylene, aromatic, methyl, and methoxy protons, respectively, as confirmed by ^13^C NMR data (Figure S7). A hydroxyl proton resonance was detected at δ 4.81 ppm.

Two-dimensional HSQC-DEPT experiments assigned the methylene signal at δ 3.91 ppm to an O-CH_2_ group (Figure S8). Upon normalization of this signal to an integral value of two, the total proton count derived from the ^1^H NMR spectrum corresponded to 22 hydrogen atoms, in agreement with the molecular formula. Combined NMR (^1^H, ^13^C, COSY, NOESY, HSQC, and HMBC) and HRMS data unambiguously confirmed the presence of a benzyl moiety linked to a terpene framework via an alkyl bridge. Key long-range and through-space correlations (Table 1) observed in the HMBC spectrum (Figure S9) and NOESY spectrum (Figure S10) substantiated this benzyl–terpene connectivity. Notably, all NMR signals of FAH-01 were assigned and confirmed for an earlier proposed structure (Lasswell and Hufford [6]; Anam et al. [7]) as 2-(5-isopropyl-4-methoxy-2-methylbenzyl)phenol.

No additional high-intensity signals were detected in the ^1^H NMR spectrum (see Figure S6), and TLC, GC, HPLC, and HRMS analyses consistently showed a single component, confirming the chemical purity of our isolate, which was subsequently used for the assays described below.

### 2.2. Anti-Contractility Effects of FAH-01 on Noradrenaline- and Phenylephrine-Induced Contractions of Human Prostate Tissues

**Figure 2:**
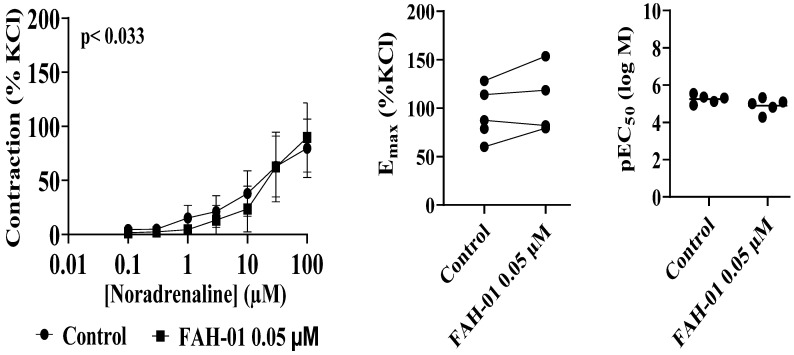
Effects of FAH-01 from *A. smeathmannii* extract on noradrenaline-induced contraction of human prostate smooth muscle tissue. Results are expressed as means ± SD (*n* = 5 patients per series, with tissue from each patient split between the FAH-01 and control groups). Tensions have been expressed as % of high molar KCl-induced contraction assessed prior to application of the control and FAH-01. E_max_ and pEC_50_ were calculated by curve fitting for each experiment.

FAH-01 produced a significant, concentration-dependent inhibition of noradrenaline (NA)-induced contractions in human prostate smooth muscle (*p* = 0.0332) across the tested NA concentrations (0.1–10 µM; Figure 2). In the presence of 0.05 µM FAH-01, NA concentration–response curves were markedly attenuated, with maximal inhibition of up to 72% observed at 1 µM NA. Specifically, contractile responses were reduced by approximately 65% at 0.1 µM NA, 51% at 0.3 µM NA, 72% at 1 µM NA, 37% at 3 µM NA, and 37% at 10 µM NA, relative to KCl-induced reference contractions. At the highest NA concentration tested (10 µM), a modest increase in the contractile response (+13%) was observed in the presence of FAH-01.

Analysis of the agonist potency revealed a reduction in the pEC_50_ values in the presence of 0.05 µM FAH-01. For example, the pEC_50_ values decreased from 5.354 (95% CI: 5.510–5.194) to 5.001 (5.151–4.840), from 5.118 (5.332–4.889) to 4.281 (4.734–3.828), from 4.924 (5.374–4.298) to 4.809 (5.413–4.205), and from 5.543 (5.839–5.242) to 5.324 (5.665–4.963), indicating a rightward shift in the NA concentration–response curves.

**Figure 3:**
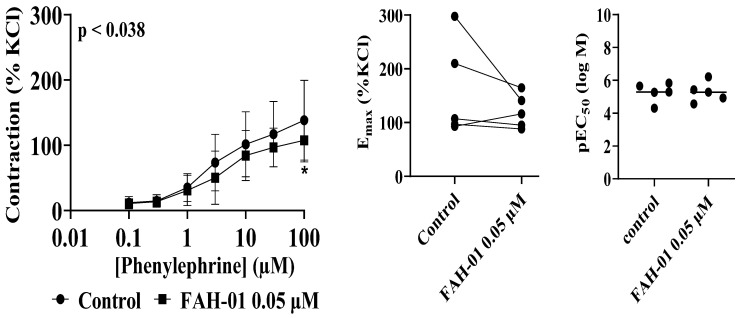
Effects of FAH-01 from *A. smeathmannii* extract on phenylephrine-induced contraction of human prostate smooth muscle tissue. Results are expressed as means ± SD (*n* = 5 patients per series, with tissue from each patient split between the FAH-01 and control groups). Tensions have been expressed as % of high molar KCl-induced contraction assessed prior to application of the control and FAH-01. * *p* < 0.05 when compared with control ethanol group. E_max_ and pEC_50_ were calculated by curve fitting for each experiment.

Similarly, FAH-01 (0.05 µM) significantly inhibited phenylephrine (PHE)-induced contractions in human prostate tissues (*p* = 0.0375) (Figure 3). Inhibition of contractile responses reached up to 63% at 0.3 µM PHE and remained substantial across higher concentrations. Specifically, contractile inhibition of approximately 50%, 45%, 38%, 37%, 38%, and 29% was observed at 1, 3, 10, 30, and 100 µM PHE, respectively, relative to KCl-induced contractions. In contrast to NA, the pEC_50_ values for PHE were not significantly altered by FAH-01, indicating suppression of maximal contractile responses without a change in agonist potency.

### 2.3. Anti-Contractility Effects of FAH-01 on Electrical Field Stimulation-Induced Contraction of Human Prostate Tissues

**Figure 4:**
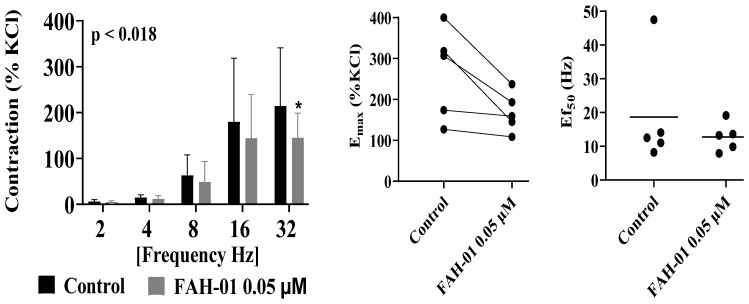
Effects of FAH-01 from *A. smeathmannii* extract on EFS-induced prostate smooth muscle contraction. Results are expressed as means ± SD (*n* = 5 patients per series, with tissue from each patient split between the FAH-01 and control groups). Tensions have been expressed as % of high molar KCl-induced contraction assessed prior to application of the control ethanol and FAH-01. * *p* < 0.05 when compared with control ethanol group. E_max_ and Ef_50_ were calculated by curve fitting for each experiment.

The addition of FAH-01 (0.05 µM) modulated electrical field stimulation (EFS)-induced contractile responses in human prostate smooth muscle across stimulation frequencies ranging from 2 to 32 Hz (Figure 4). At lower stimulation frequencies, FAH-01 produced inhibitory effects, with contractile responses being reduced by approximately 21% at 2 Hz, 21% at 4 Hz, 23% at 8 Hz, 23% at 16 Hz, 20% at 24 Hz, and 20% at 32 Hz, relative to KCl-induced reference contractions.

In contrast, at higher stimulation frequencies (8–32 Hz), FAH-01 was associated with modest increases in EFS-induced contractions, reaching approximately 22% at 8 Hz, 16% at 16 Hz, and 10% at 32 Hz. These bidirectional effects suggest frequency-dependent modulation of neurogenic contractility.

Analysis of the effective stimulation frequency (EF_50_) values revealed alterations in the presence of FAH-01. At 2 Hz, the EF_50_ values shifted from 1.042 (95% CI: 0.972–1.113) under control conditions to 0.994 (0.989–1.321) with FAH-01. At 4 Hz, the EF_50_ values changed from 0.912 (0.998–1.422) to 0.898 (0.861–0.935). At higher frequencies, the EF_50_ values decreased from 1.677 (1.412–1.942) to 1.281 (1.134–1.428) at 16 Hz and from 1.148 (1.133–1.163) to 1.134 (1.106–1.162) at 32 Hz in the presence of FAH-01.

Collectively, these findings indicate that FAH-01 modulates neurogenic contractile responses in human prostate smooth muscle in a frequency-dependent manner.

### 2.4. Acute Toxicity in Artemia salina

Administration of FAH-01 to third-instar larvae of *A. salina* resulted in an estimated median lethal concentration (LC_50_) of 19.85 µM after 24 h of exposure across the tested concentration range (1–50 µM). In contrast, the negative controls—aqueous NaCl (6%, *w/v*) and ethanol (1%)—produced mortality rates of 0% and 1.67%, respectively, whereas the positive control, potassium dichromate (K_2_Cr_2_O_7_, 1%), induced 100% mortality (Figure S11).

### 2.5. DPPH Activity

FAH-01 has an IC_50_ of 0.546 µg/mL (95% CI: 0.341–0.7351 µg/mL) versus 0.342 µg/mL (95% CI: 0.3081–0.373 µg/mL) in the gallic acid standard agent against the DPPH radical-scavenging assay (Figure S12).

## 3. Discussion

This study reports the isolation, structural characterization, and biological evaluation of 2-(5-isopropyl-4-methoxy-2-methylbenzyl)phenol (FAH-01; chamanen), a benzyl–terpene hybrid compound obtained from the roots of *Acridocarpus smeathmannii*. We found no match for FAH-01 in the NIST mass spectral library, despite previous reports of the compound (chamanen), which likely reflects the limited database coverage of rare natural products. Although FAH-01 has been previously reported, its chemical structure has not been fully characterized in publicly accessible spectral libraries, which explains the lack of a corresponding NIST entry and highlights the added value of the present work. Importantly, structural identification in this study was not based on library matching alone, but was supported by comprehensive spectroscopic characterization, including 1D and 2D NMR and high-resolution mass spectrometry. Thus, FAH-01 was purified using repeated preparative chromatographic techniques and its structure was unambiguously established by comprehensive spectroscopic analysis, including two-dimensional NMR, RP-HPLC, and GC-HRMS. Given that FAH-01 has only been sparsely documented in the literature [6,7], the present study solidifies the existing knowledge by confirming its structure using modern analytical techniques. It expands our knowledge about its natural occurrence in *A. smeathmannii*.

Structurally, FAH-01 represents a covalently linked benzyl–terpene hybrid, integrating two pharmacologically relevant motifs within a single molecular framework. Terpene scaffolds are well-known for their ability to interact with biological membranes, enzymes, and receptors, often conferring favorable lipophilicity and three-dimensional conformational properties that enhance target selectivity [8,9]. In parallel, benzyl moieties contribute hydrophobic interactions, π–π stacking capability, and chemical stability, while also permitting synthetic derivatization for medicinal chemistry optimization [11]. The coexistence of these features in FAH-01 may underlie its observed bioactivity and support the concept that hybrid molecules can display enhanced structure–activity relationships compared with their individual components [9,17].

In functional assays, FAH-01 demonstrated pronounced inhibitory effects on adrenergic- and neurogenic-mediated contractions in human prostate smooth muscle. The compound attenuated noradrenaline- and phenylephrine-induced contractions and modulated electrical field stimulation-evoked neurogenic responses, indicating interference with both receptor-dependent and nerve-mediated contractile mechanisms. As prostatic smooth muscle tone is primarily regulated by sympathetic adrenergic signaling [18], these findings position FAH-01 as a potential modulator of pathological smooth muscle hypercontractility. The dual inhibition of adrenergic agonist responses and neurogenic output suggests a multimodal mechanism of action, which may be advantageous in conditions such as benign prostatic hyperplasia and lower urinary tract symptoms, where treatment resistance remains clinical [19]. In this study, it is believed that the hybrid benzyl–terpene structure of FAH-01 may enable simultaneous interaction with receptor binding domains and lipid membranes, thereby facilitating both receptor-level antagonism and the modulation of ion channel activity.

Regarding the potential mechanisms underlying biphasic responses, we found that FAH-01 (Chamanen), isolated and purified from roots of *A. smeathmannii*, exerts inhibitory effects on human prostate smooth muscle contractility, with evidence suggesting a biphasic, concentration-dependent response. This characteristic indicates that multiple mechanisms are likely to be engaged, both in the concentration and the physiological context of stimulation. A primary mechanism underlying the observed effects may involve interference with α_1_-adrenoceptor-mediated signaling, which plays a central role in regulating prostate smooth muscle tone. Following a lower concentration, FAH-01 appears to attenuate contractile responses, which is consistent with the functional antagonism of adrenergic stimulation. This aligns with the established therapeutic strategy for benign prostatic hyperplasia (BPH), where α_1_-adrenoceptor blockers reduce smooth muscle tone to enhance urinary flow. The inhibitory action observed suggests that FAH-01 may act, at least in part, through a similar pathway. In addition to receptor-level effects, the modulation of intracellular calcium dynamics likely contributes significantly to the biphasic response. Smooth muscle contraction depends on increases in intracellular Ca^2+^, primarily through voltage-gated calcium channels and receptor-operated pathways [20]. For instance, FAH-01 may reduce calcium influx at lower concentrations, thereby suppressing contraction. However, at higher concentrations, the compound may engage additional targets, including intracellular calcium release mechanisms or calcium sensitization pathways, potentially altering the magnitude or nature of the response. Such dual possible modulation of calcium handling may be a well-recognized basis for biphasic pharmacological effects in smooth muscle systems. On the other hand, this condition may contribute to the regulation of potassium channels. The activation of potassium channels leads to membrane hyperpolarization and reduced excitability of smooth muscle cells [21]. FAH-01 may promote potassium efflux at lower concentrations, reinforcing its relaxant effect. At higher concentrations, however, desensitization of these channels or interaction with other ion transport mechanisms could diminish this effect or introduce competing influences, thereby contributing to the biphasic pattern. This duality could explain shifts in the contractile responses due to FAH-01, possibly showing partial agonism or mixed pharmacological activity. The reduction in maximal responses without consistent shifts in agonist potency suggests that FAH-01 may act predominantly as a non-competitive or functional antagonist, rather than a purely competitive inhibitor.

Furthermore, non-specific effects at higher concentrations cannot be excluded. Due to its lipophilic terpene structure, FAH-01 may interact with cell membranes, altering the membrane fluidity and indirectly affecting receptor conformation and ion channel function. These non-selective interactions may become more prominent at higher doses and contribute to deviations from the initial inhibitory response. We cannot rule out the important role of adaptive cellular mechanisms whereby prolonged or high-dose exposure to FAH-01 may trigger compensatory signaling pathways that may trigger cascades of intracellular processes. These findings position FAH-01 as a lead compound for the development of novel agents targeting lower urinary tract symptoms associated with BPH. However, more studies are required to delineate its precise molecular targets, characterize its receptor binding profile, and evaluate its efficacy and safety in vivo. Our observed increase in contraction at higher noradrenaline concentrations may reflect partial agonistic activity or differential receptor engagement at elevated agonist levels. We hope that understanding the mechanisms underlying its biphasic behavior will be critical for advancing its pharmacological development and ensuring predictable clinical outcomes.

FAH-01 also demonstrated antioxidant activity in the DPPH radical-scavenging assay, which is consistent with reports that benzylated phenolics and oxygenated terpenes can modulate oxidative stress [10]. While this assay provides only a preliminary indication of antioxidant capacity, the results align with the compound’s predicted interactions with oxidative and inflammatory regulators and support its potential relevance in oxidative stress-related pathologies. The antioxidant activity of FAH-01 may complement its anti-contractile effects by mitigating the oxidative stress-induced enhancement of smooth muscle tone.

The *Artemia salina* assay provides only a preliminary indication of general cytotoxicity and does not reliably predict mammalian toxicity, pharmacokinetics, or organ-specific adverse effects. However, it provides a suitable guide for early-stage toxicity information. Nevertheless, further evaluation using mammalian cell lines and in vivo models is necessary to accurately determine the compound’s safety profile, pharmacokinetic behavior, and potential organ-specific toxicities. From our results, acute toxicity assessment using *Artemia salina* larvae revealed moderate toxicity at micromolar concentrations, providing an initial safety profile for FAH-01. Aquatic bioassays are widely employed as early indicators of general toxicity and developmental interference prior to vertebrate studies [22]. Although such assays do not directly predict mammalian toxicity, they offer valuable guidance for subsequent preclinical evaluations and dose selection.

This study has limitations. For instance, the precise molecular mechanisms underlying the anti-contractile effects of FAH-01 were not elucidated. The relatively small sample size represents a limitation and may affect the statistical robustness of our findings. However, studies with larger patient cohorts are required to confirm reproducibility. A key limitation of this study is the absence of direct investigations into the underlying molecular mechanisms. Specifically, radioligand binding assays were not performed to determine the affinity of FAH-01 for α_1_-adrenoceptor subtypes (α_1A_, α_1B_, α_1D_). In addition, intracellular calcium dynamics were not assessed by using calcium imaging techniques. Furthermore, functional interaction studies employing selective antagonists, such as prazosin (α_1_-adrenoceptor blocker) or nicardipine (L-type Ca^2+^ channel blocker), were not conducted. Furthermore, the biological activities were evaluated primarily in vitro, necessitating confirmation in relevant in vivo models.

In our next step, we aim to ensure continuity and thus enhance translational relevance. This would address the current limitations, particularly the absence of receptor-level characterization, intracellular signaling data, and in vivo validation, and provide a clearer pathway from ex vivo findings to therapeutic application. Firstly, we are extending our anti-contractility study to graded doses across different tissues, including smooth muscle of the prostate and bladder, and porcine arteries. Also, detailed receptor pharmacology would be prioritized and explored. Since the current data suggest interference with adrenergic signaling but do not distinguish between receptor subtypes, radioligand binding assays using human recombinant α1-adrenoceptor subtypes would allow for determination of the binding affinity and selectivity profile of FAH-01. Additional experiments on functional assays in heterologous expression systems would complement binding studies by letting us know whether FAH-01 could act as a competitive antagonist, a non-competitive antagonist, or a partial agonist. Furthermore, a comparative assessment of selective antagonists with different subtypes in organ bath experiments would be used to evaluate subtype-specific contributions to the observed anti-contractile effects.

Secondly, FAH-01 would be applied to studies on intracellular signaling and ion channel modulation. Also, calcium imaging experiments using Fluo-4 AM in prostate smooth muscle cells would help to clarify whether FAH-01 reduces intracellular Ca2+ influx or release. Patch-clamp electrophysiology would further be used to evaluate its effects on L-type Ca^2+^ channels and various potassium channels, which are central to smooth muscle excitability. Pharmacological modulation with agents such as nicardipine (L-type Ca^2+^ channel blocker) or tetraethylammonium (K^+^ channel blocker) would help to confirm these pathways.

Thirdly, we hope that expanding to relevant human and animal cell models will improve the translational depth. Experiments using primary human prostate stromal cells, smooth muscle cells, and epithelial cells would allow for the assessment of cytotoxicity, antiproliferative effects, and phenotype modulation by comparing contractile versus synthetic states. In parallel, established cell lines such as WPMY-1 (stromal) would be applied for higher-throughput screening. These studies would help us to determine whether FAH-01 exerts disease-modifying effects beyond acute smooth muscle relaxation, particularly in the context of benign prostatic hyperplasia.

Fourthly, in vivo validation is essential to bridge the gap to clinical translation. Suitable models, including testosterone-induced or surgically induced BPH in rodents, where endpoints such as the prostate weight, prostate-specific antigen level, urinary flow parameters, and histological changes can be assessed, are underway. Pharmacokinetic indices would also be conducted to determine the bioavailability and systemic exposure. Importantly, dose-ranging toxicity studies in mammals would be used to complement the preliminary *Artemia salina* assay, which only provides limited predictive value for human safety.

Furthermore, medicinal chemistry and structure–activity relationship studies would enhance the drug development potential of FAH-01. Given its hybrid benzyl–terpene scaffold, systematic modification of the aromatic and terpene moieties could optimize the potency, selectivity, and pharmacokinetic properties. Testing these analogs across the aforementioned receptor, cellular, and in vivo systems would provide a coherent framework linking the chemical structure of FAH-01 to its biological function. These could help to establish a mechanistic continuum from receptor binding to whole-organism efficacy, thereby significantly strengthening the translational trajectory of FAH-01 as a potential therapeutic agent for LUTS and associated disorders.

Conclusively, Chamanen emerges as a bioactive benzyl–terpene hybrid with potent inhibitory effects on adrenergic and neurogenic contraction in human prostate smooth muscle, supporting its therapeutic potential in conditions associated with increased smooth muscle tone. Further studies that aim to elucidate its mechanisms of action, toxicity screening, and in vivo efficacy are essential.

## 4. Materials and Methods

### 4.1. Chemicals

The adrenergic agonists, noradrenaline and phenylephrine, were purchased from Sigma-Aldrich (Munich, Germany). *n*-Hexane and deuterated chloroform (CDCl_3_) were also obtained from Sigma-Aldrich (Munich, Germany). All other solvents and reagents that were used were of an analytical grade.

### 4.2. Plant Collection and Authentication

Details of plant collection, voucher specimen deposition (FHI 113685), research approval for the use of *Acridocarpus smeathmannii* roots, and processing into powdered material have been reported previously [23]. In addition, a phytosanitary certificate (No. 0124876) was issued by the Nigerian Agricultural Quarantine Service, Plant Health Division.

### 4.3. Extraction and Liquid–Liquid Fractionations

Briefly, powdered roots of *Acridocarpus smeathmannii* (2.2 kg) were extracted with *n*-hexane (EMPLURA^®^), using a Soxhlet apparatus, for 4 h. The solvent was removed under reduced pressure, using a rotary evaporator (IKA^®^ RV 10D S93) coupled to a Vacuubrand CVC 3000 vacuum controller (IKA^®^, RV 10D S93, Baden-Wurttemberg, Germany) at 40 ± 1 °C and 120 mbar. The resulting light-golden yellow extract (6.36 *w*/*w*) was reconstituted, stored at 4 °C, and aliquoted for subsequent purification and in vitro biological assays.

### 4.4. Chromatography Studies

Preparative thin-layer chromatography (TLC) was performed on pre-coated silica gel 60 F_254_ and cellulose plates (Merck, Darmstadt, Germany). The plate visualization was carried out under UV light at 365 nm (Jeulin^®^ Enceinte, model 701435) (468 Rue Jacques Monod, Évreux, France).

For preparative separation, column chromatography of 20 cm × 5 cm glass column was packed with silica gel 60 (35–75 µm particle size; CAS No. 7631-89-9, Merck KGaA, Darmstadt, Germany). The column was sealed and overlaid with a 3 mm layer of sea sand (Cat. No. 1313710000, Grüssing GmbH, Filsum, Germany) to protect the stationary phase.

A comprehensive structural elucidation of FAH-01 was accomplished using serial TLC, two-dimensional NMR spectroscopy, high-performance liquid chromatography (HPLC), gas chromatography–mass spectrometry (GC-MS), and high-resolution mass spectrometry (HRMS).

### 4.5. Gas Chromatography–Mass Spectrometry (GC-MS) and High-Resolution Mass Spectrometry Analysis (HR-MS)

Gas chromatography–mass spectrometry (GC-MS) analysis was performed as previously reported [4]. Briefly, a solution of FAH-01 (30 µg in 500 µL chloroform) was prepared. A fraction of 1 µL was injected in 1:5 split mode at an injector and interface temperature of 260 °C. A temperature gradient (50 °C for 3 min and 50–310 °C with 10 °C/min) was applied with a helium inlet pressure of 70 kPa at the beginning. Data were collected in scan mode using “LabSolution” software-GCMS-Q2010 Plus (Shimadzu^®^; Kyoto, Japan).

Additionally, high-resolution mass spectrometry was done using a Trace GC Ultra (Thermo Scientific, Waltham, MA, USA) with a direct inlet system and EI ionization.

### 4.6. Nuclear Magnetic Resonance Analysis

Nuclear magnetic resonance (NMR) spectroscopy was performed using 5 mg of FAH-01 dissolved in 500 µL of deuterated chloroform (CDCl_3_). ^1^H NMR spectra were acquired at 27 °C on a Bruker Avance III 500 MHz spectrometer (Bruker, Ettlingen, Germany) equipped with a 5 mm SEI inverse probe (^1^H/^13^C, Z-gradient). Two-dimensional NMR experiments, including COSY, HSQC, HMBC, and NOESY, were recorded using standard Bruker pulse sequences with TOPSPIN software (version 3.5). Spectral processing and analysis were conducted using MNova software (version 15.0.1).

### 4.7. Reverse-Phase High-Performance Column Chromatography (RP-HPLC)

High-performance liquid chromatography (HPLC) was performed on a Shimadzu system (Shimadzu^®^ Europa GmbH, Duisburg, Germany) equipped with an LC-10AS pump, SIL-20A HT autosampler, and SPD-10AV UV–Vis detector. Separations were carried out on a LiChrospher^®^ RP-18 column (5 µm particle size, 160 mm × 4.6 mm) (Merck, Darmstadt, Germany). FAH-01 aliquots (5 µL) were diluted in methanol to a final volume of 1.5 mL, transferred to glass vials, and injected into the HPLC system.

The column was operated under isocratic conditions, using 60% aqueous methanol as the mobile phase at a flow rate of 0.5 mL/min. Elution was monitored by UV detection at 280 nm.

### 4.8. Biological Actions of Isolate on Human Prostate Tissue Contraction

Organ bath experiments were performed using periurethral prostate tissue obtained from patients undergoing radical prostatectomy (rPx) for prostate cancer, as previously described [4,5,24]. The tissue samples were processed, and the experiments were initiated within 1 h of surgical removal. Preparations were mounted in organ baths and stretched to an initial resting tension of approximately 4.9 mN, followed by a 45 min equilibration period. During equilibration, spontaneous decreases in tone were observed; therefore, the tension was readjusted up to three times, until a stable resting tone of 4.9 mN was achieved.

After equilibration, maximal contractile capacity was assessed by stimulation with 80 mM KCl. Tissues were then washed three times with Krebs–Henseleit (KH) solution, after which FAH-01 or vehicle (ethanol, control) was added. Cumulative concentration–response curves for noradrenaline (NA) and phenylephrine (PHE) were generated 30 min after the addition of FAH-01 or ethanol. From each patient, tissue samples were divided into control and FAH-01 groups (two preparations per group) and analyzed as paired samples within the same experiment. Different patient samples were used for each agonist-induced contraction series; thus, the group sizes were identical within each experimental series.

Agonist-induced contractions were expressed as percentages of the maximal contraction elicited by 80 mM KCl (phasic peak), to account for inter-individual variability in stromal/epithelial composition, smooth muscle content, degree of benign prostatic hyperplasia, and other tissue heterogeneities [4,5]. A final concentration of 0.05 µM FAH-01 was achieved in a 10 mL organ bath and compared with the ethanol control group.

The maximum contractile responses (E_max_), concentrations producing 50% of the maximal agonist-induced contraction (EC_50_), and frequencies producing 50% of the maximal electrically evoked contraction (Ef_50_) were calculated for each individual experiment by nonlinear curve fitting, using GraphPad Prism (GraphPad Software Inc., San Diego, CA, USA). EC_50_ values were expressed as negative logarithms of the molar concentration (pEC_50_) to quantify the agonist potency [4].

This study received experimental approval to conduct an in vitro study using human prostate tissue from the Ethics Committee of Ludwig-Maximilians University (LMU), Munich, Germany (LMU/MH060922). The in vitro experiments involving human prostate tissues were conducted in accordance with the Declaration of Helsinki of the World Medical Association and were approved by the Ethics Committee of Ludwig-Maximilians University (LMU), Munich, Germany. LMU Klinikum Munich, Germany, was responsible for obtaining informed consent from all patients (date of approval: 6 September 2022). Research permission to perform experiments with *A. smeathmannii* roots constituents on human and animal was approved by the Health Research and Ethics Committee, College of Medicine, University of Lagos (CMUL/HREC/09/18/424). Additionally, the authors obtained a phytosanitary certification (No. 0124876) from the Nigeria Agricultural Quarantine Service Plant Health, Nigeria. 

### 4.9. Effects of Isolates on the Prostate Smooth Muscle Contractile Activity

The modulatory effects of an equimolar concentration of FAH-01 on human prostate tissue contractility were evaluated and compared with vehicle-treated controls. Contractile responses were elicited using the non-selective adrenergic agonist noradrenaline (NA; 0.1–100 µM) and the α_1_-adrenoceptor-selective agonist phenylephrine (PHE; 0.1–100 µM). The anti-contractile effects of FAH-01 were expressed as positive percentage changes, relative to the contractile response induced by each agonist alone, normalized to the maximal contraction produced by 80 mM KCl, as previously described [4].

### 4.10. Electrical Field Stimulation

Electrical field stimulation (EFS) was applied to generate the frequency–response curves of the neurogenically induced contractions 30 min after the addition of FAH-01 or vehicle control (1% ethanol) [4]. EFS evokes action potentials leading to the release of endogenous neurotransmitters, including noradrenaline and acetylcholine. Tissue strips were mounted between two parallel platinum electrodes connected to a CS4 stimulator (Danish Myotechnology, Hinnerup, Denmark). Square-wave pulses (monophasic) with a pulse width of 1 ms and an amplitude of 20 V were delivered in stimulation trains. The contractile responses were recorded at stimulation frequencies of 2, 4, 8, 16, and 32 Hz, with a 60 s interval between successive stimulations. Only one frequency–response curve was obtained per tissue sample. EFS-induced contractions were quantified by measuring peak contraction amplitudes and expressed as percentages of the maximal phasic contraction elicited by 80 mM KCl. The maximum responses (E_max_) and frequencies inducing 50% of the maximal EFS response (Ef_50_) were determined by nonlinear curve fitting, using GraphPad Prism.

### 4.11. In Vitro Antioxidant Assay

The antioxidant activity of FAH-01 was assessed using the 2,2-diphenyl-1-picrylhydrazyl (DPPH) radical-scavenging assay, as previously described [25]. Briefly, a 0.1 mM DPPH solution was prepared in methanol and protected from light. FAH-01, the reference standard gallic acid, and the control were dissolved in methanol at the required concentrations. Aliquots (1.5 mL) of each sample or gallic acid standard were mixed with 1.5 mL of the DPPH solution and incubated for 30 min at room temperature in the dark. The absorbance was subsequently measured at 517 nm.

### 4.12. Acute Toxicity Potential in Artemia salina

*A. salina* (brine shrimp) cysts were obtained from JBL GmbH & Co. KG (Neuhofen, Germany) and decapsulated in aqueous NaCl solution (6%, *w*/*v*). Approximately 1.5 g of cysts were incubated in 1 L of aerated saline in a transparent glass hatching vessel at 29 ± 1 °C under continuous illumination (1200 lx) to promote larval development [26]. Strong aeration was maintained throughout incubation. After 48 h, larvae were collected and separated to obtain third-instar (instar III) larvae (0.45–0.8 mm in length) [27].

For the acute toxicity assay, 30 instar III larvae were transferred into each well of 48-well plates (in triplicate), containing 1 mL of FAH-01 at concentrations ranging from 0.1 to 50 µM. The control groups included saline water, ethanol (1%), and potassium dichromate (K_2_Cr_2_O_7_, 1%). Plates were incubated at room temperature with gentle shaking under an 18:10 h light/dark cycle, and larvae were not fed during the exposure period. After 24 h, mortality was assessed by counting larvae that showed no movement upon observation using a VHX digital microscope (KEYENCE HX-970F; Keyence Corporation, Osaka, Japan).

### 4.13. Data and Statistical Analyses

Results from concentration–response and frequency–response curves, as well as zones of inhibition, are presented as mean ± standard deviation (SD). For the organ bath experiments, post hoc multiple comparisons at individual agonist concentrations or stimulation frequencies were performed using two-way ANOVA with appropriate multiple-comparison corrections. Each experimental series comprised *n* = 5 independent experiments, including paired samples analyzed within each experiment. Statistical analyses were conducted using GraphPad Prism version 9.5.0 (GraphPad Software Inc., San Diego, CA, USA). The sample size (*n* = 5 per experimental series) was selected based on prior organ bath studies and pilot experiments demonstrating sufficient power to detect ≥ 30% differences in contractile responses with acceptable variability.

Maximum contractile responses (E_max_), agonist potencies (pEC_50_), and frequencies producing 50% of the maximal EFS-induced contraction (Ef_50_) were calculated as mean values for each experimental series and compared between groups using paired *t*-tests, as previously described [28]. Changes in concentration and frequency–response relationships are reported as percentage differences, relative to the control values (mean difference [MD] with 95% confidence intervals, normalized to KCl-induced contractions).

In acute toxicity experiments, median lethal doses (LD_50_) were estimated by probit analysis, using log–dose response curves constructed in GraphPad Prism. The LD_50_ values were calculated from triplicate measurements at each concentration.

## Figures and Tables

**Table 1:** ^1^H and ^13^C NMR data of 2-(5-isopropyl-4-methoxy-2-methylbenzyl)phenol in CDCl_3_.

Atom Position	^13^C Chemical Shift in ppm	Predicted ^13^C Chemical Shift in ppm	CH_3_, CH_2_, CH, or C	^1^H Chemical Shift in ppm	HH Coupling Constant in Hz	^1^H Signal Integral	HH COSY Correlation to H#	HH NOESY Correlation to H#	HMBC from Indexed C to H#
P1	153.85	155.3	C						P5, P3, P6
P2	126.71	128.2	C						P4, P6, OH, P7
P3	130.25	130.7	CH	6.93 (d)	7.7	1	P7 (w)	6	P5, P7
P4	120.72	121.8	CH	6.84 (t)	7.7	1		P5	P6
P5	127.44	127.6	CH	7.12 (t)	7.7	1		P4	P3
P6	115.49	116.4	CH	6.79 (d)	7.7	1			P4, OH
P7	33.59	32.9	CH_2_	3.91 (s)		2	6 (w), P3 (w)	6, OH, 7	6
1	135.22	131.0	C						6, P7, 7
2	128.81	134.2	C						3, P7, 7
3	112.98	113.3	CH	6.68 (s)		1		11, 7	7
4	155.48	155.4	C						3, 6, 11, 8
5	134.70	135.2	C						3, 8, 9, 10
6	127.44	128.1	CH	6.96 (s)		1	P7 (w), 7 (w)	P7, P3, 9, 10	P7, 8, 7
7	19.56	19.4	CH_3_	2.22 (s)		3	6 (w)	3, P7	3, 6
8	26.56	27.6	CH	3.24 (sep)	6.9	1	9, 10	9, 10	6, 9, 10
9	22.70	23.6	CH_3_	1.15 (d)	6.5	3	8	6, 8	8
10	22.70	23.6	CH_3_	1.15 (d)	6.5	3	8	6, 8	8
11	55.43	56.1	CH_3_	3.81 (s)		3		3	
OH				4.81		1		P7	

## Data Availability

Data is contained within the article.

## Supplementary Materials

The following supporting information can be downloaded at: https://www.mdpi.com/article/10.3390/molecules31091380/s1. Figure S1: Gas Chromatogram and MS analysis of FAH-01 from *A. smeathmannii* root extracts (M^+^ at m/z 270.16 and a base peak at m/z 107; Figure S2: Suggested fragmentations leading to the observed mass spectrum of FAH-01; Figure S3: HRMS output of FAH-01; Figure S4: Spectrometry analysis of HPLC chromatograms of FAH-01 from *A. smeathmannii* root extracts (aqueous MeOH, 60:40, 270 nm). Figure S5: TLC of FAH-01; Figure S6. ^1^H NMR spectrum of FAH-01 at 500 MHz. The solvent was CDCl_3_; Figure S7. ^13^C NMR spectra of FAH-01 at 125 MHz. Trace 1, ^1^H decoupled ^13^C spectrum; trace 2: DEPT135 spectrum with the CH_2_ signal in negative phase, CH_3_ and CH in positive phase; trace 3, DEPT90 with CH signals only; Figure S8. Multiplicity-edited HSQC spectrum with the CH_2_ in negative phase (blue). CH_3_ and CH signals are displayed in positive phase (red); Figure S9. HMBC spectrum of FAH-01; Figure S10. NOESY spectrum of FAH-01. NOE signals are displayed in negative phase (blue); Figure S11: Acute Toxicity effect of FAH-01 in third larval developmental stage of *A. salina*; Figure S12: In vitro antioxidant activity of FAH-01 against DPPH (garlic acid standard.

## Abbreviations

Distortionless enhancement by polarization transfer (DEPT); Gas chromatography–mass spectrometry (GC-MS); Heteronuclear multiple bond correlation (HMBC); Heteronuclear single quantum coherence (HSQC); High-resolution mass spectrometry (HRMS); Homonuclear correlation spectroscopy (COSY); Negative logarithms of the molar concentration for agonists (pEC_50_); Noradrenaline (NA); Nuclear magnetic resonance (NMR); Nuclear overhauser effect spectroscopy (NOESY); Phenylephrine (PHE); Radical prostatectomy (rPx); Retention factor (R_f_); Thin layer chromatography (TLC).
