# Supplementary material for: A Bioactive Benzyl Terpene from Acridocarpus smeathmannii Inhibits Human Prostate Smooth Muscle Contractility
Source: Molecules. 2026 Apr 22;31(9):1380. doi: 10.3390/molecules31091380 (PMC13165309; doi:10.3390/molecules31091380)
Supplement: Supplementary file 1 [file molecules-31-01380-s001.zip › molecules-4185946-supplementary.pdf]

Supplementary:

A bioactive benzyl terpene from Acridocarpus smeathmannii inhibits human prostate smooth muscle contractility.

Oluwafemi Ezekiel Kale, Claudia Huber, Denis Schuldeis, Alexander Tamalunas, Martin Hennenberg and Wolfgang Eisenreich

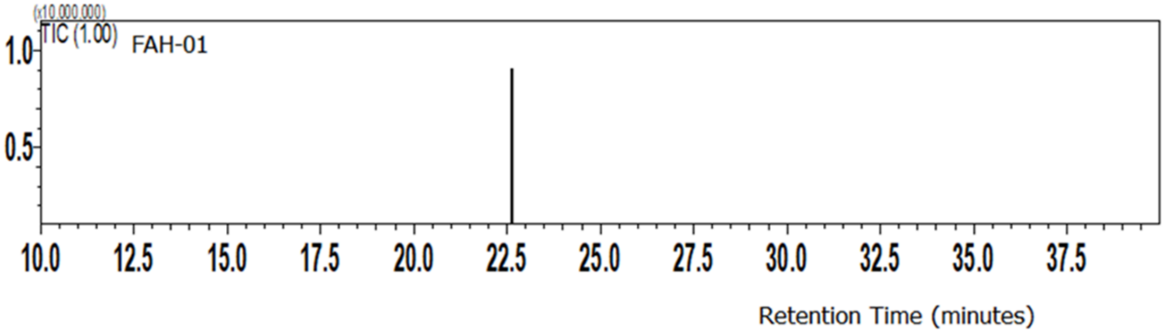

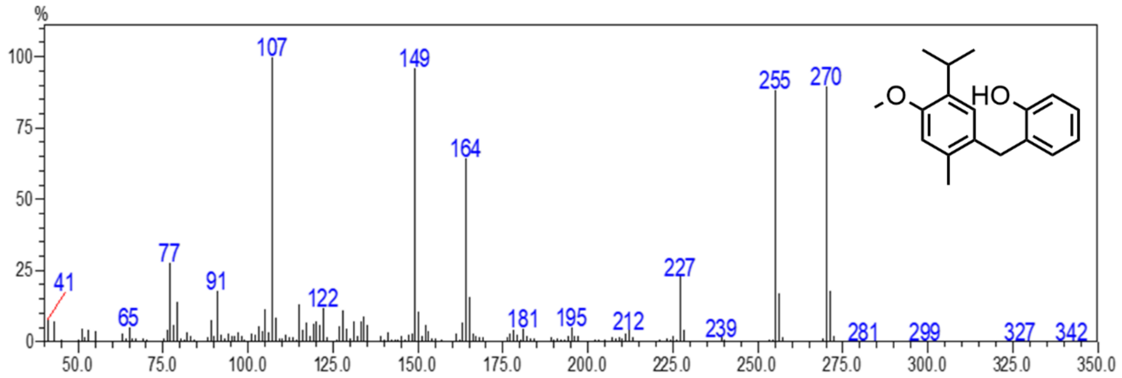

Figure S1: Gas Chromatogram and MS analysis of FAH-01 from *A. smeathmannii* root extracts (M^+ at m/z 270.16 and a base peak at m/z 107).

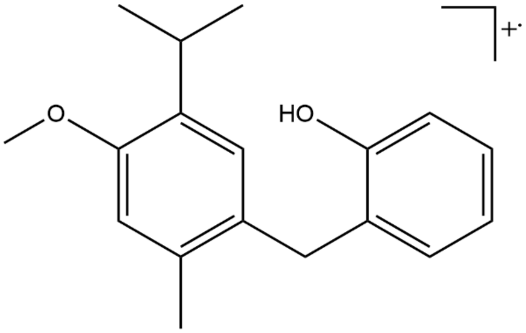

Chemical Formula: $C_{18}H_{22}O_2$
Exact Mass: 270,16

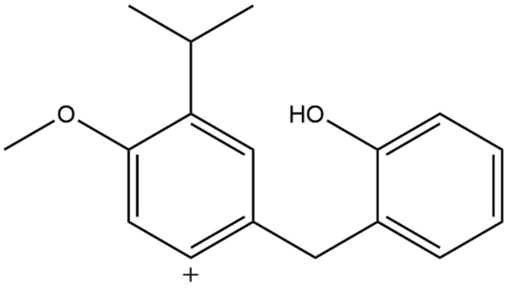

Chemical Formula: $C_{17}H_{19}O_2^+$
Exact Mass: 255,14

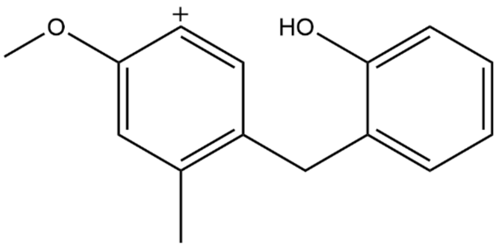

Chemical Formula: $C_{15}H_{15}O_2^+$
Exact Mass: 227,11

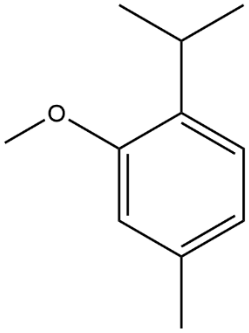

Chemical Formula: $C_{11}H_{16}O$
Exact Mass: 164,12

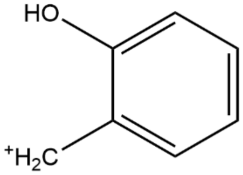

Chemical Formula: $C_7H_7O^+$
Exact Mass: 107,05

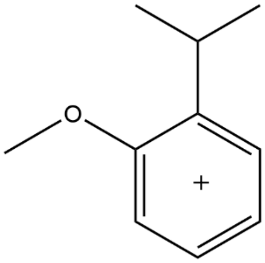

Chemical Formula: $C_{10}H_{13}O^+$
Exact Mass: 149,10

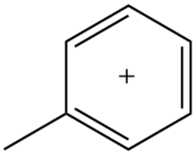

Chemical Formula: $C_7H_7^+$
Exact Mass: 91,05

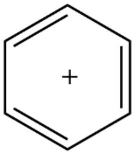

Chemical Formula: $C_6H_5^+$
Exact Mass: 77,04

Figure S2: Suggested fragmentations leading to the observed mass spectrum of FAH-01.

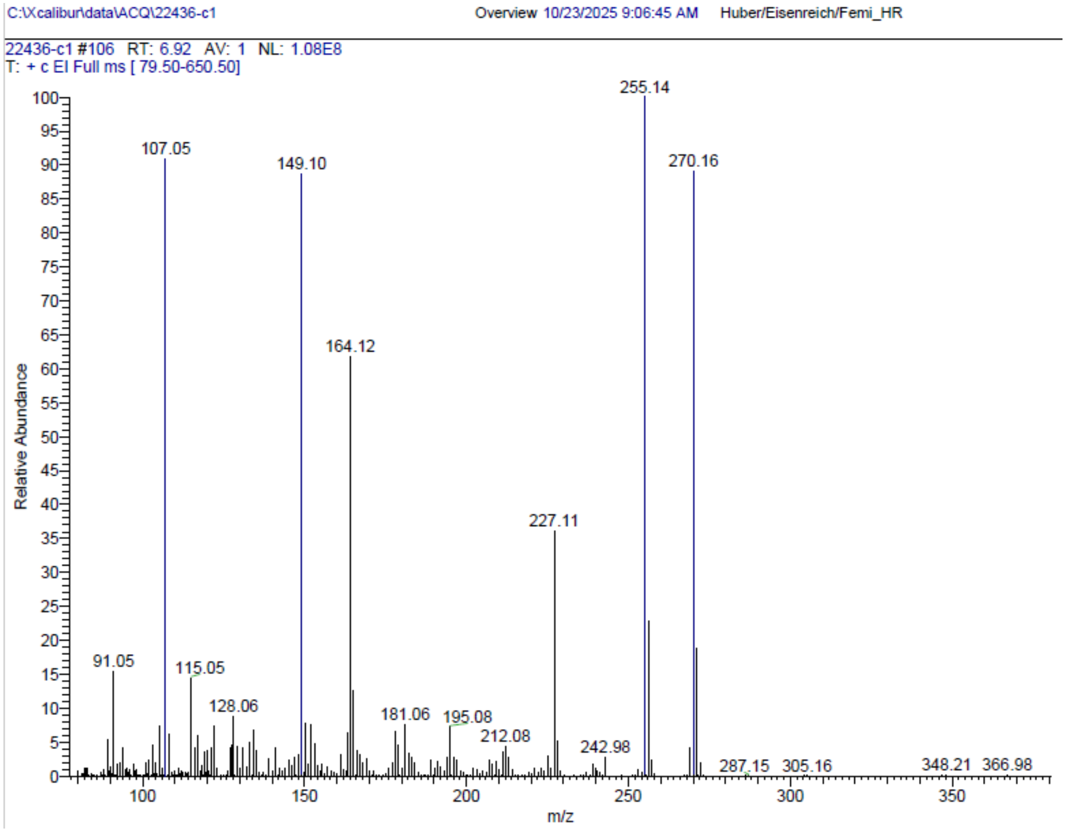

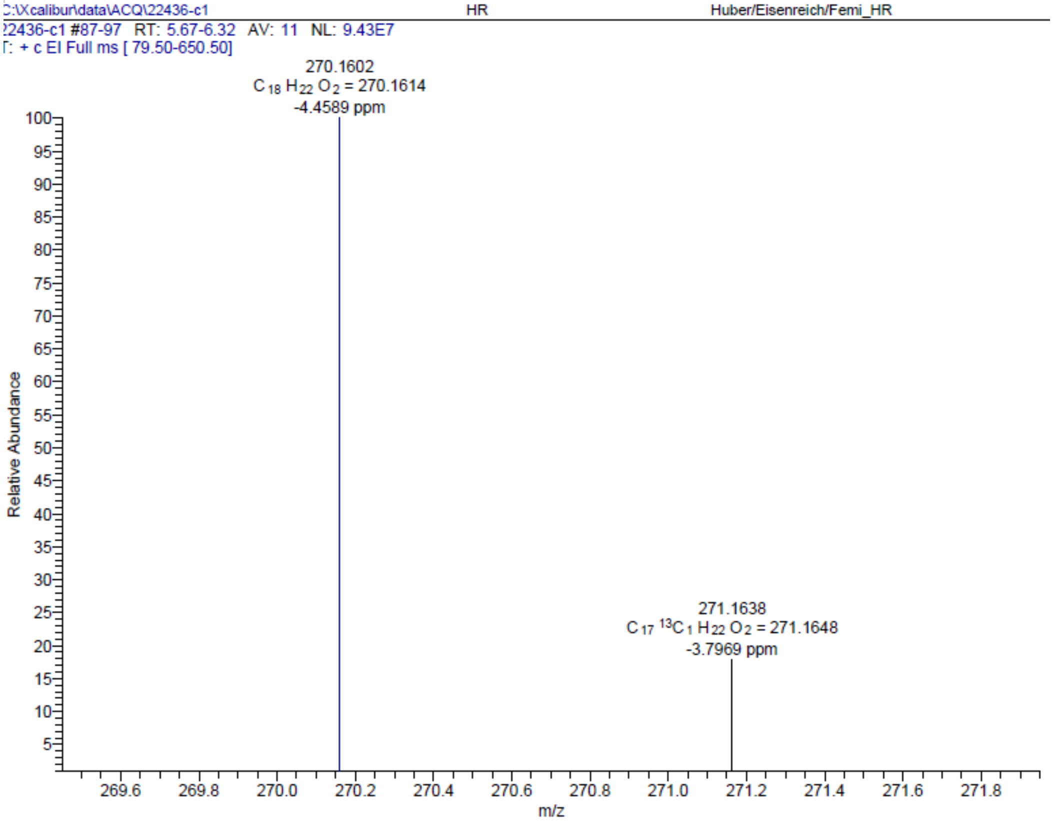

Figure S3: HRMS output of FAH-01

18.11.2025 18:20 Chromatogram C:\Clarity Lite\DataFiles\Flavine\Data\FMBB64LH_06.10.2025 16_46_17_072.PRM Page 1 of 2

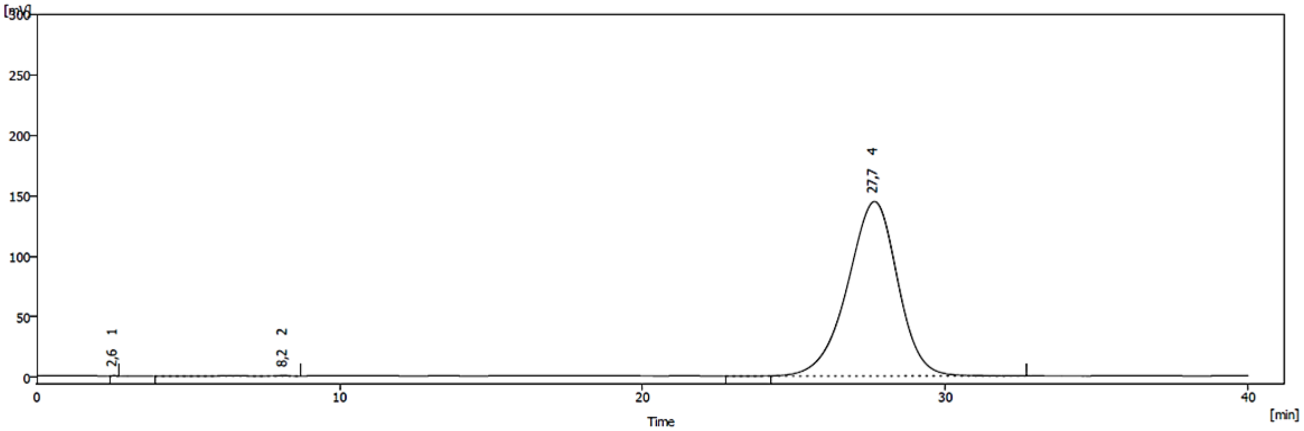

All Signals Result Table (Uncal - FMBB64LH_06.10.2025

	Signal	Signal Name	Reten. Time [min]	Start Time [min]	End Time [min]	Apex Value [mV]	Start Value [mV]	End Value [mV]	Area [mV.s]	Height [mV]	Area [%]	Height [%]	W05 [min]	Compound Name
1	1	Detector 1	2,552	2,404	2,720	1,461	0,952	0,906	3,884	0,530	0,0	0,4	0,12	
2	1	Detector 1	8,156	3,908	8,700	1,480	0,867	0,959	27,933	0,531	0,2	0,4	0,44	
3	1	Detector 1	24,211	22,757	24,251	1,221	0,934	0,962	4,954	0,260	0,0	0,2	0,16	
4	1	Detector 1	27,661	24,251	32,681	145,288	0,962	1,122	16658,453	144,261	99,8	99,1	1,75	
			All Signals Total						16695,224	145,582	100,0	100,0		

Figure S4: Spectrometry analysis of HPLC chromatograms of FAH-01 from *A. smeathmannii* root extracts (aqueous MeOH, 60:40, 270 nm).

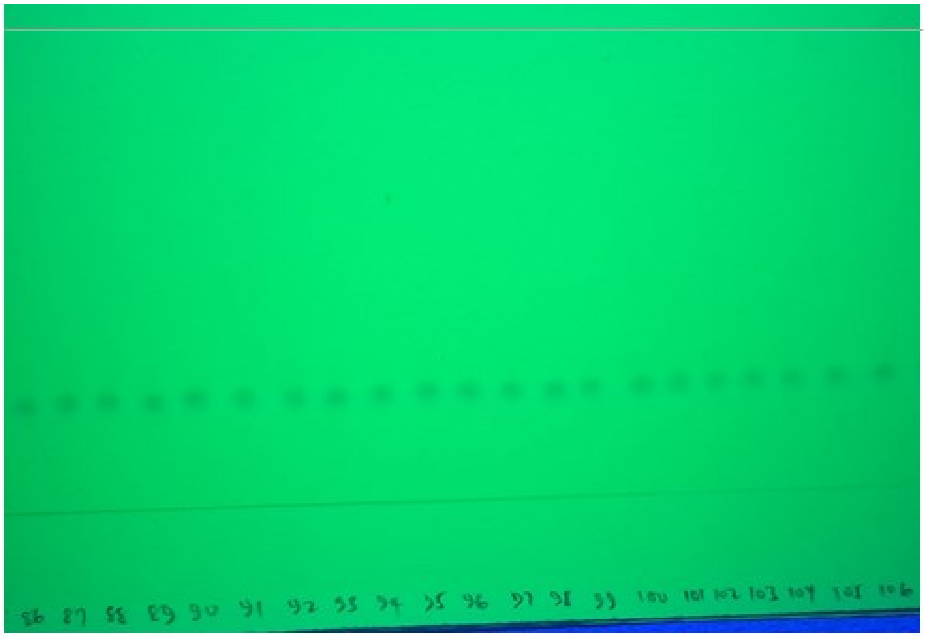

Figure S5: TLC of FAH-01

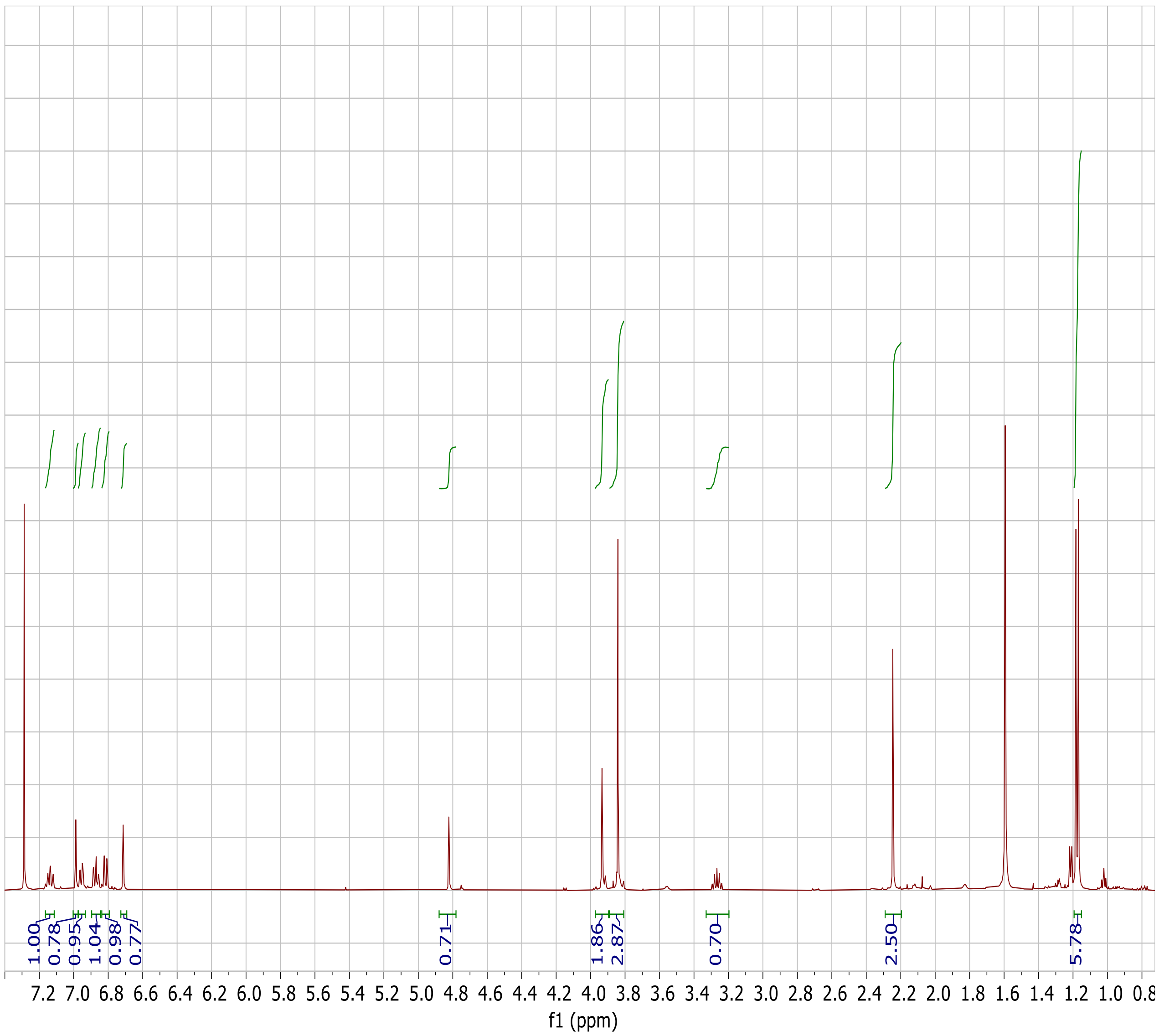

Figure S6. ^{1}H NMR spectrum of FAH-01 at 500 MHz. The solvent was $CDCl_3$.

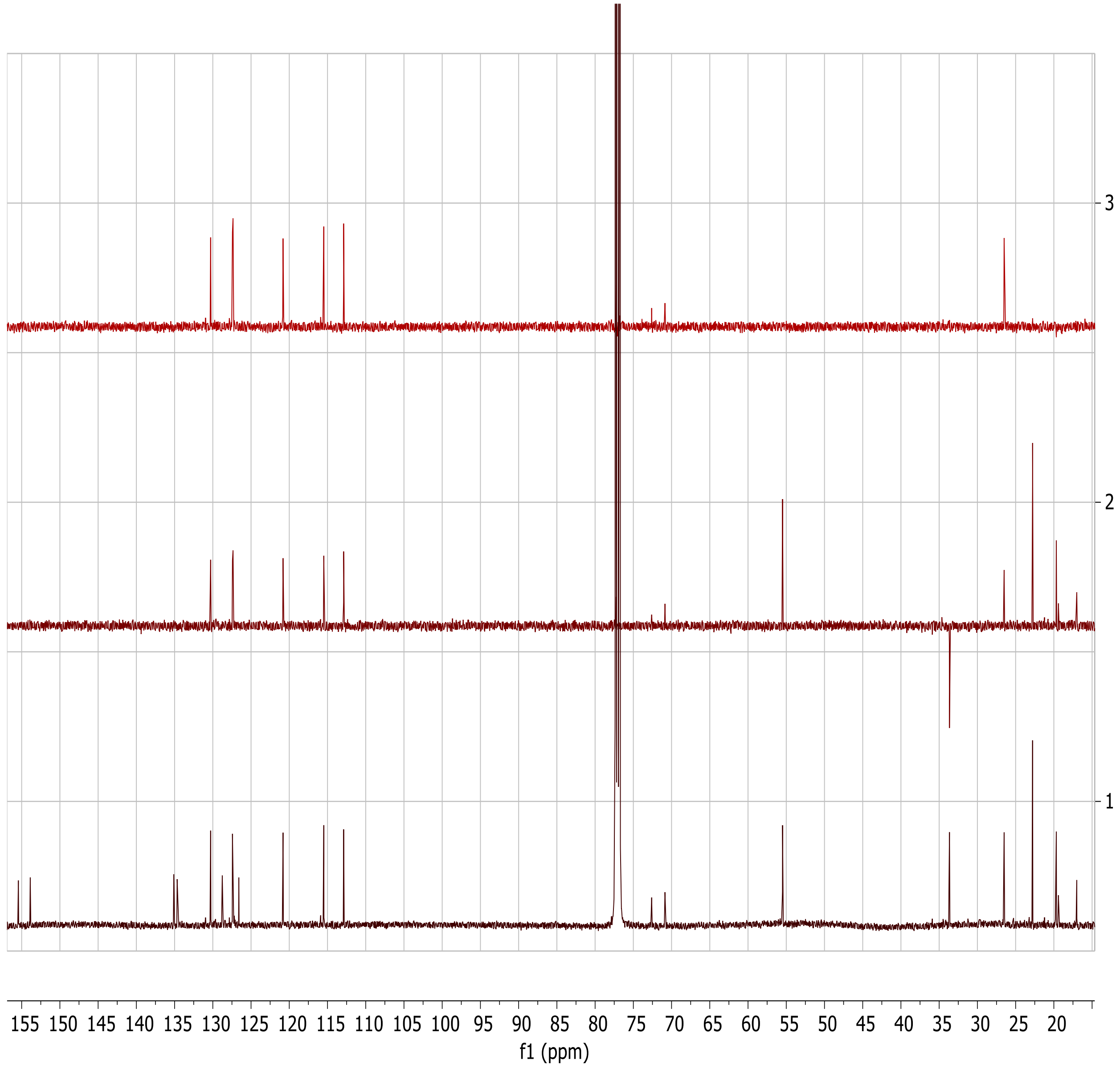

Figure S7. ^{13}C NMR spectra of FAH-01 at 125 MHz. Trace 1, ^{1}H decoupled ^{13}C spectrum; trace 2: DEPT135 spectrum with the CH_2 signal in negative phase, CH_3 and CH in positive phase; trace 3, DEPT90 with CH signals only

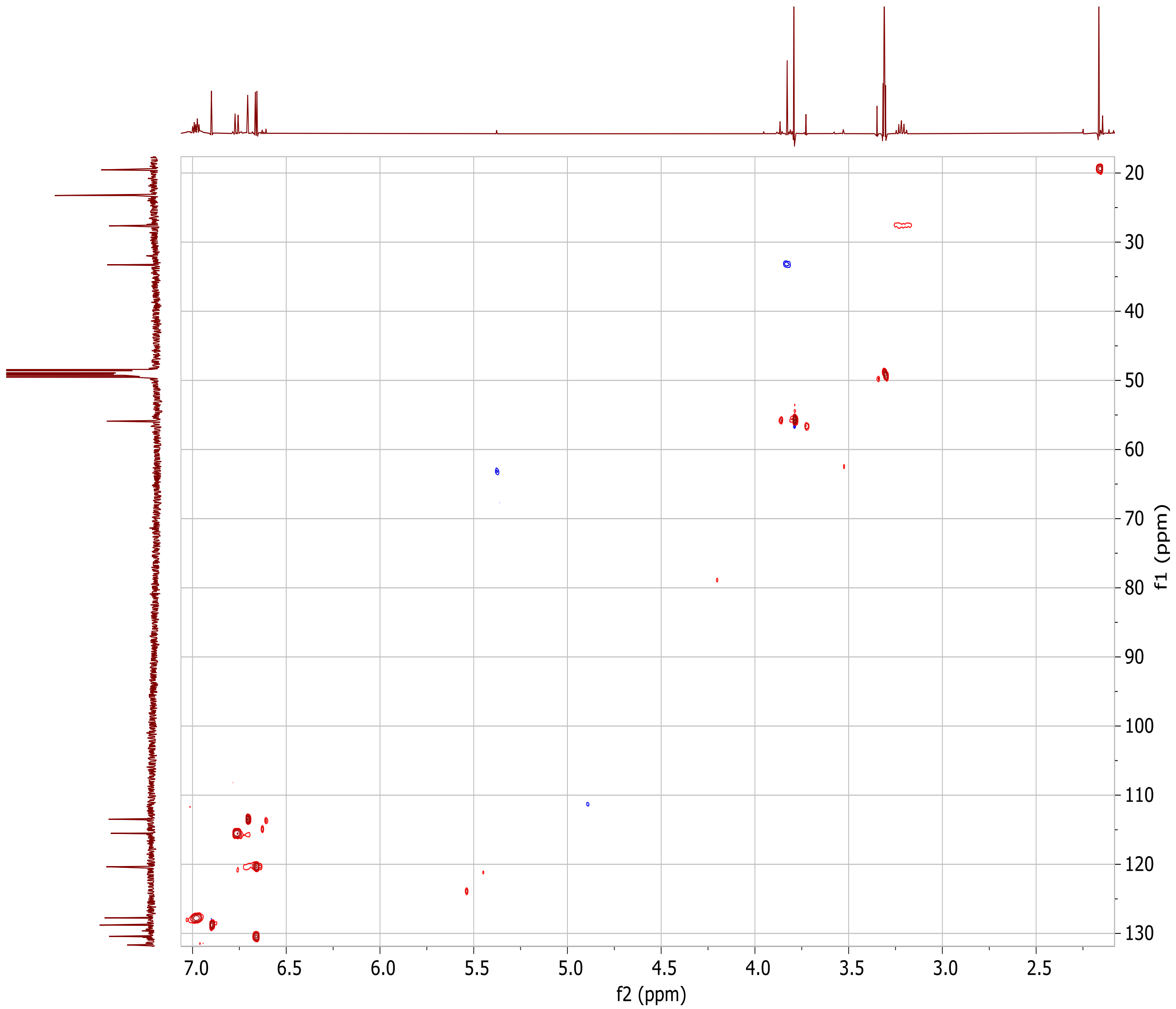

Figure S8. Multiplicity-edited HSQC spectrum with the CH_2 in negative phase (blue). CH_3 and CH signals are displayed in positive phase (red)

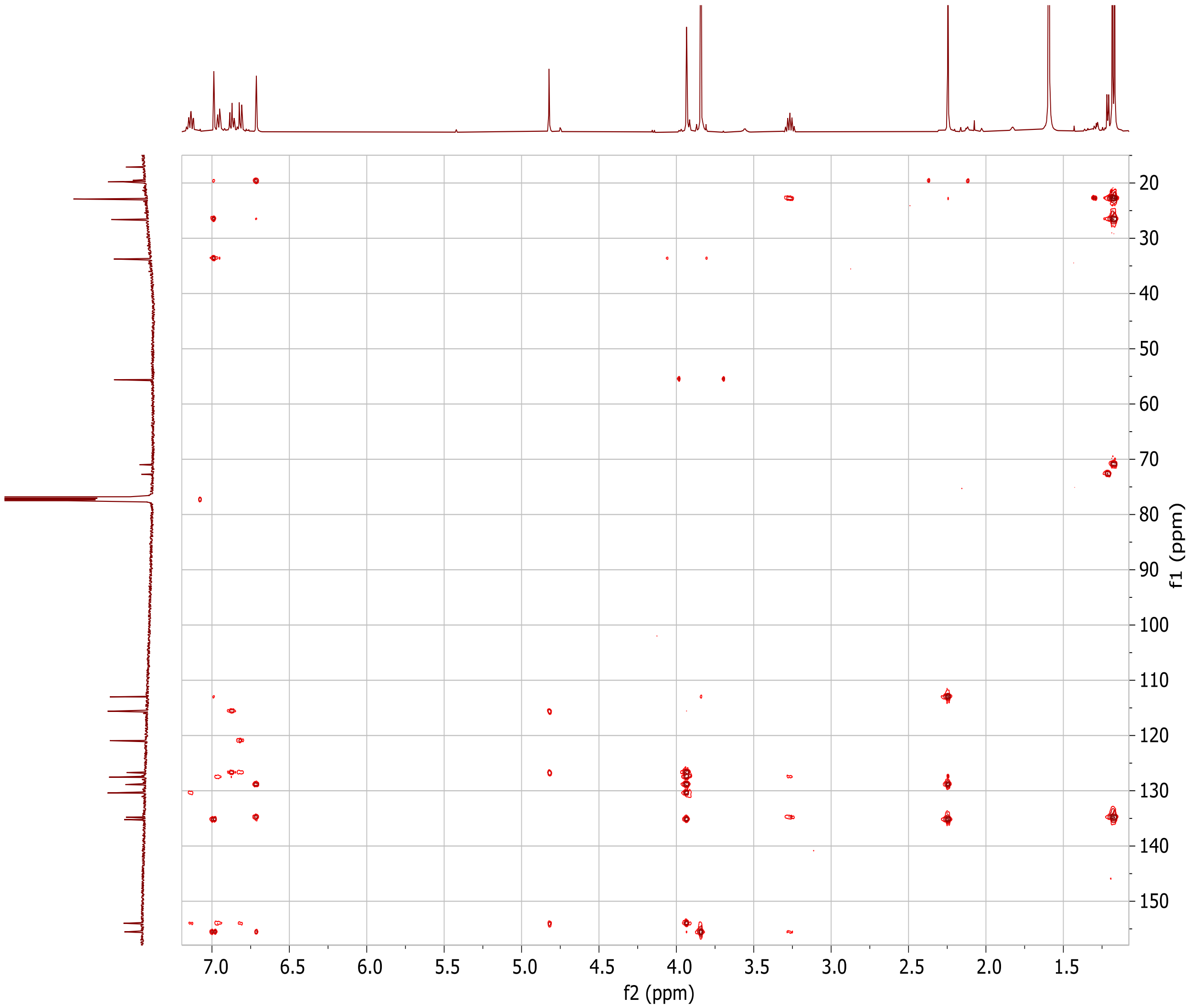

Figure S9. HMBC spectrum of FAH-01

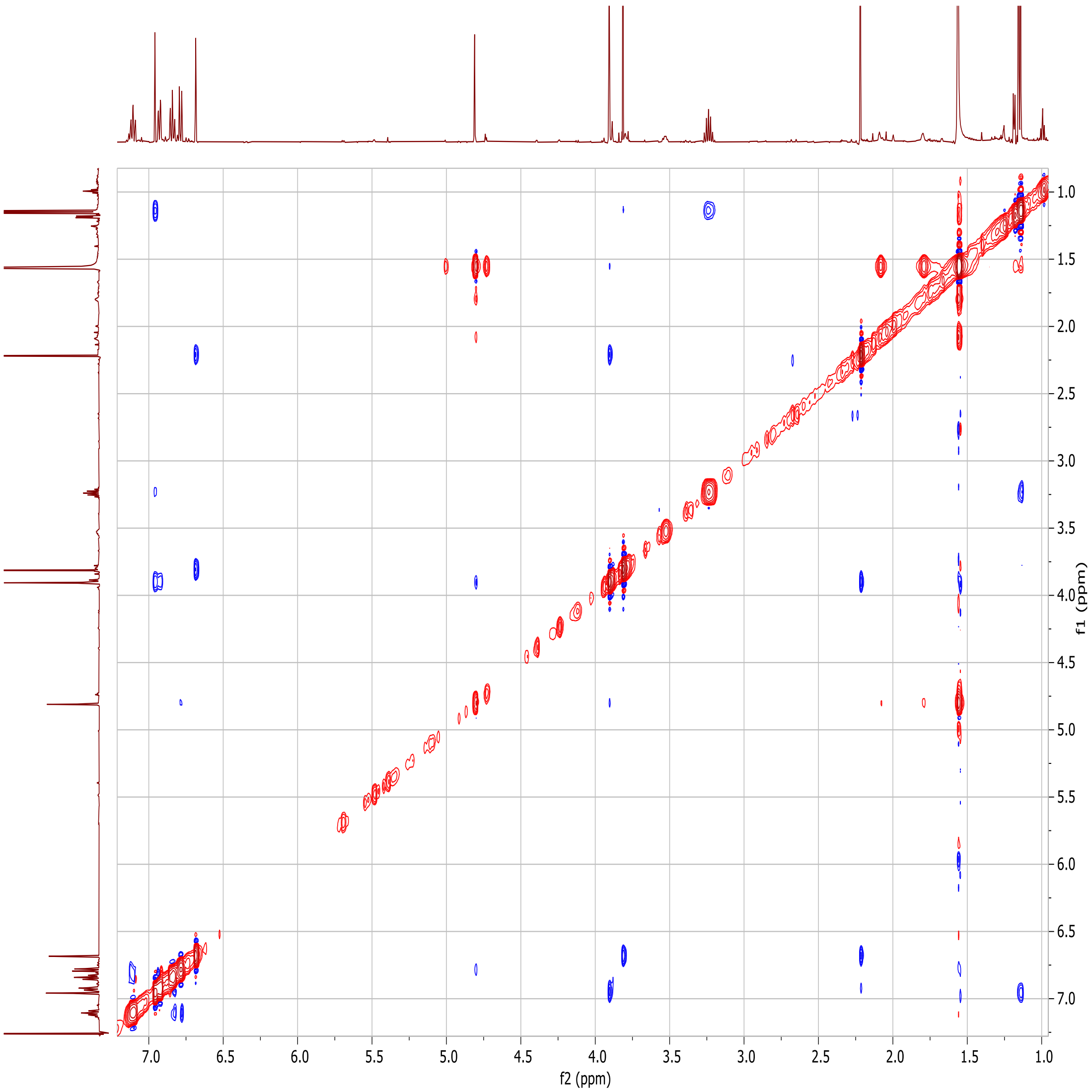

Figure S10. NOESY spectrum of FAH-01. NOE signals are displayed in negative phase (blue)

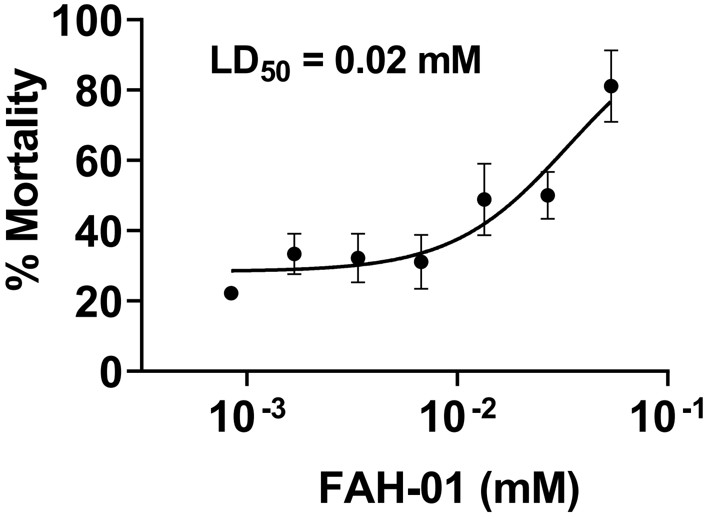

Figure S11: Acute Toxicity effect of FAH-01 in third larval developmental stage of *A. salina*

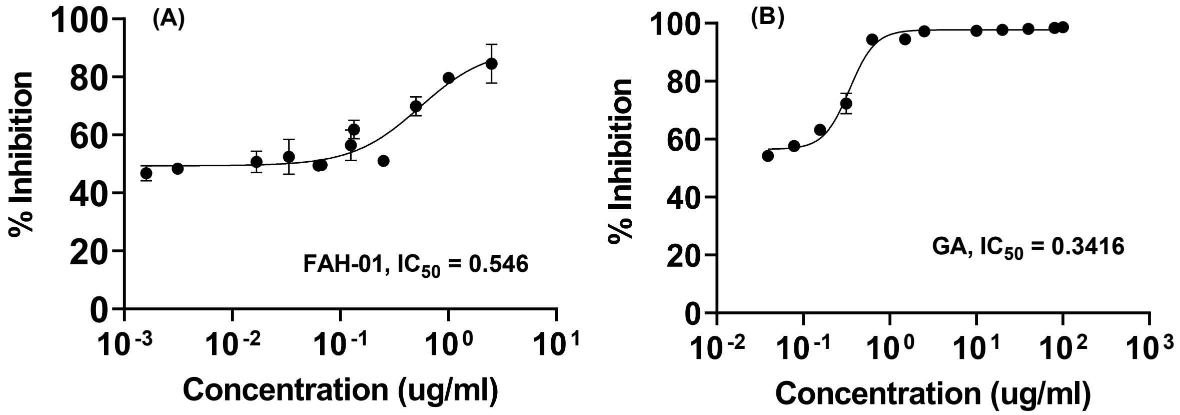

Figure S12: In vitro antioxidant activity of FAH-01 against DPPH (garlic acid standard).
